# Preparation of a Cellulose Column for Enhancing the Sensing Efficiency of the Biocide 2-n-Octyl-4-Isothiazolin-3-One

**DOI:** 10.3390/polym12112712

**Published:** 2020-11-16

**Authors:** Seong Ho Hong, Sang Wook Kang

**Affiliations:** 1 Department of Chemistry, Sangmyung University, Seoul 03016, Korea; wkn7836@daum.net; 2 Department of Chemistry and Energy Engineering, Sangmyung University, Seoul 03016, Korea

**Keywords:** cellulose acetate (CA), 2-n-Octyl-4-Isothiazolin-3-one (OIT), porous membrane, water-pressure, hydrophobic

## Abstract

In this study, a cellulose acetate (CA) membrane with pores generated by a water pressure treatment was investigated for its ability to serve as a pretreatment filter device for the detection of 2-n-octyl-4-isothiazolin-3-one (OIT). Pores were generated by applying a water pressure of 8 bar to a membrane manufactured using a CA-based polymer solution. The CA used for the manufacturing was an environment-friendly, low-cost and highly energy-efficient material. Furthermore, since the fabricated porous CA polymeric film possessed many hydrophilic functional groups, it could strongly bind hydrophilic substances while avoiding interaction with hydrophobic substances. OIT, which comprises a hydrophobic bond that forms weak bonds over time, can break down more easily than hydrophilic impurities. The different extents of interaction occurring between either the toxic fungicide OIT or the hydrophilic impurities and the CA film were determined by Fourier-transform infrared (FT-IR) spectroscopy. The physicochemical changes in the resulting membrane, which occurred when the pores were generated, were investigated through scanning electron microscopy (SEM) and thermogravimetric analysis (TGA).

## 1. Introduction

Porous materials offer many advantages, such as high surface area, energy efficiency, high permeability and low weight [1,2,3,4,5,6]. Therefore, they have been widely applied for the development of batteries, separation and filtration materials, catalysts for synthesis and heat dissipation sensors, among other applications [7,8,9,10,11,12,13,14,15,16,17].

In a recent study, a porous polybenzimidazole (PBI) membrane was used as a battery separator. This PBI membrane contained numerous amine groups that enabled dendrite-free Li deposition, while exhibiting a stable and low overpotential cycling durability. Furthermore, in order to increase the voltage efficiency, 2,3-epoxypropyltrimethylammonium chloride (EPTMA-Cl) was used as a grafting agent to synthesize the PBI membranes grafted with quaternary ammonium groups [18,19].

In the sensing field, Alrammouza et al. developed a sensor that integrated a graphene oxide detection layer with a paper substrate. This graphene-oxide-based sensor displayed good response to humidity due to the presence of many pores and a high hydrophobicity [20]. Zhang et al. synthesized a super-porous NiFe_2_O_4_ microsphere structure and used it to manipulate a sensor. The porous NiFe_2_O_4_ microspheres were highly sensitive and selective for detecting acetone over other volatile organic gases [21]. Tan et al. produced an optical sensor using a metal–organic framework as a porous material. This photo-responsive adsorbent underwent adsorption and desorption in response to remote stimuli, introduced porous materials and allowed for potential energy conversion applications [22]. Rahman et al. detected 3-methoxyphenol (3-MP) using a chemical sensor made of a synthesized Ag_2_O/carbon black nanocomposite (CB NC) that was deposited on a glassy carbon electrode (GCE) [23]. Kopytina et al. used a tetradecylphosphonium salt to detect pertechnetate ions. A sensor based on a polyvinyl chloride (PVC) polymeric matrix membrane successfully quantified pertechnetate ions in radioactive waste [24]. Hierarchical porous Au/ZnO microspheres with a diameter of 10–12 μm were used as a CH_4_ gas sensor. An Au/ZnO-based sensor with an Au content of 1.0% exhibited a maximum detection performance of 4.16 at 100 ppm of CH_4_ and 250 °C [25].

In the gas separation field, Liu et al. employed a polyvinyl-alcohol-polysulfone (PVA-PS) composite membrane for the separation of a CO_2_/N_2_ mixed gas [26]. Neha Tyagi et al. suggested a graphene-based ultra-thin film for gas separation. Furthermore, these nanoporous graphene (NPG) membranes have potential applications in halogen separation [27]. Lei Wang et al. generated a membrane using a synthesized porous aromatic framework-97 (PAF-97) through a one-step, acid-catalyzed reaction. PAF-97 increased the structural flexibility due to the presence of ether functional groups. The selectivity of this membrane for CO_2_/CH_4_ was 22.48 [28]. Anna Ovchrova et al. proposed an ethylene/ethane gas separation process based on polysulfone. Ethylene has a permeability of 185 L for this membrane. This value was the best result achievable in the porous literature [29].

Soesantoa et al. investigated the performance of powdered activated-carbon dynamic membranes (PAC DMs) for high-efficiency seawater pretreatment. In the contamination mechanism, the seudosteady-state filtration flow rate and the refusal of cross-flow microfiltration was increased by 59% and 29%, respectively, when PAC DM was used than PAC DM was not used. As a result, this study provided insight into the Fenton oxidation process (FOP) cleaning technology for PAC DM crossflow cleaning, thus enabling the automation and optimization of the DM operation [30].

In this study, we propose a porous cellulose acetate (CA) membrane as a pretreatment filter for the development of 2-n-octyl-4-isothiazolin-3-one (OIT)-sensitive sensor. CA polymer-based films contain many hydrophilic moieties (e.g., ether and carbonyl groups), and hence can be expected to bind hydrophilic impurities, but not OIT. It was based on the method of fabricating a porous material using hydraulic pressure on a membrane made of CA and Ni salts proposed in our laboratory [31]. Films generated in this new way are expected to be applicable to more diverse fields. The water uptake performance of the film was measured to confirm that it could serve as a sensor by strongly adsorbing hydrophilic impurities, except OIT [32]. Fourier-transform infrared (FT-IR) spectroscopy was conducted to investigate the interaction between the membrane and either hydrophilic impurities or OIT. The produced film, which possessed many hydrophilic groups and exhibited excellent water adsorption performance, was found to be a suitable pretreatment filter for the OIT sensors.

## 2. Materials and Methods

### 2.1. Materials

CA (Mn ~50,000) was purchased from Aldrich Co. (Darmstadt, Germany). 2-n-Octyl-4-isothiazolin-3-one was purchased from Tokyo Chemical Industry Co. (Tokyo, Japan). 2-n-Octyl-4-isothiazolin-3-one is a light-yellow oil, with a molecular weight of 213.34 g/mol and a boiling point of 120 °C. Nickel(II) nitrate hexahydrate was obtained from Samchun Pure Chemical Co. (Seoul, Korea). Acetone was acquired from Daejung Chemical & Metals (Gyeonggi, Korea). All reagents were used without any treatment.

### 2.2. Membrane Fabrication

A 10% (*wt*/*wt*) CA (Mn ~50,000) solution was prepared by dissolving CA in acetone/water (*wt*/*wt* 8:2). The solution was stirred for 3 h, followed by the addition of 0.23 mol of Ni(NO_3_)_2_·6H_2_O per mol of CA, and the mixture was stirred for approximately 2 h. The prepared polymer solution was a clear, viscous, cyan liquid, and when the solvent evaporated, the polymer CA precipitated. The solution was coated on a glass plate at 200 µm, based on the solution state, using the freestanding method for membrane fabrication. Subsequently, pore formation was induced in the membrane using a water pressure treatment device at a pressure of 8 bar. The fabricated membrane was dried in a vacuum oven for 2 days.

### 2.3. Experimental

Samples of the contaminant were prepared upon dissolution in ethanol. These solutions were prepared by reducing the amount of 2-n-octyl-4-isothiazolin-3-one per repeating unit mole of CA by 1/10 from 1.0 × 10^−1^ moles. The adsorption extent was measured by FT-IR spectroscopy (VERTEX 70, BRUKER, Billerica, MA, USA).

## 3. Results

### 3.1. Water Uptake Data

**Figure 1:**
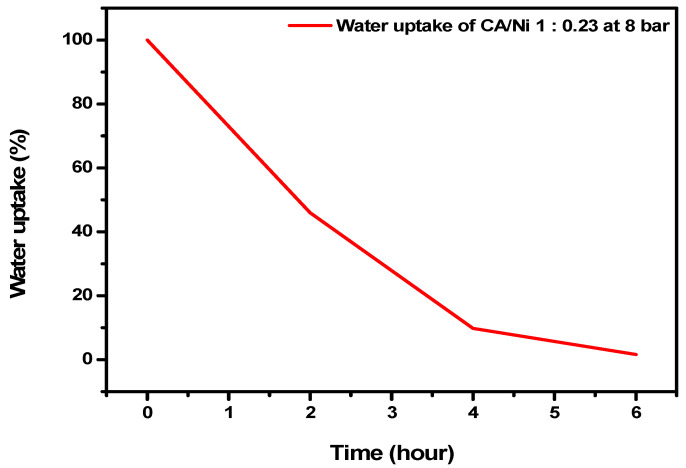
Water uptake data for CA/Ni(NO_3_)_2_·6H_2_O measured as a function of time (h).

A water uptake experiment was conducted to investigate the intensity with which the manufactured membrane interacted with the hydrophilic substances. Upon subjecting a CA/Ni(NO_3_)_2_·6H_2_O 1:0.23 membrane to an 8-bar water pressure treatment, it could be expected that the membrane would adsorb water and hydrophilic substances due to the presence of hydrophilic functional groups such as carbonyl and ether moieties. As shown in Figure 1 and Table 1, the results of this water adsorption experiment indicated that the water uptakes by the CA membrane were 45.97%, 9.75% and 1.60% after 2, 4 and 6 h, respectively. Therefore, the CA membrane fabricated using a nickel salt proved to be a highly hydrophilic membrane; consequently, OIT—a hydrophobic substance—was not well adsorbed. Therefore, this membrane may be suitable for use as a pretreatment column to adsorb hydrophilic impurities.

### 3.2. Scanning Electron Microscope (SEM)

**Figure 2:**
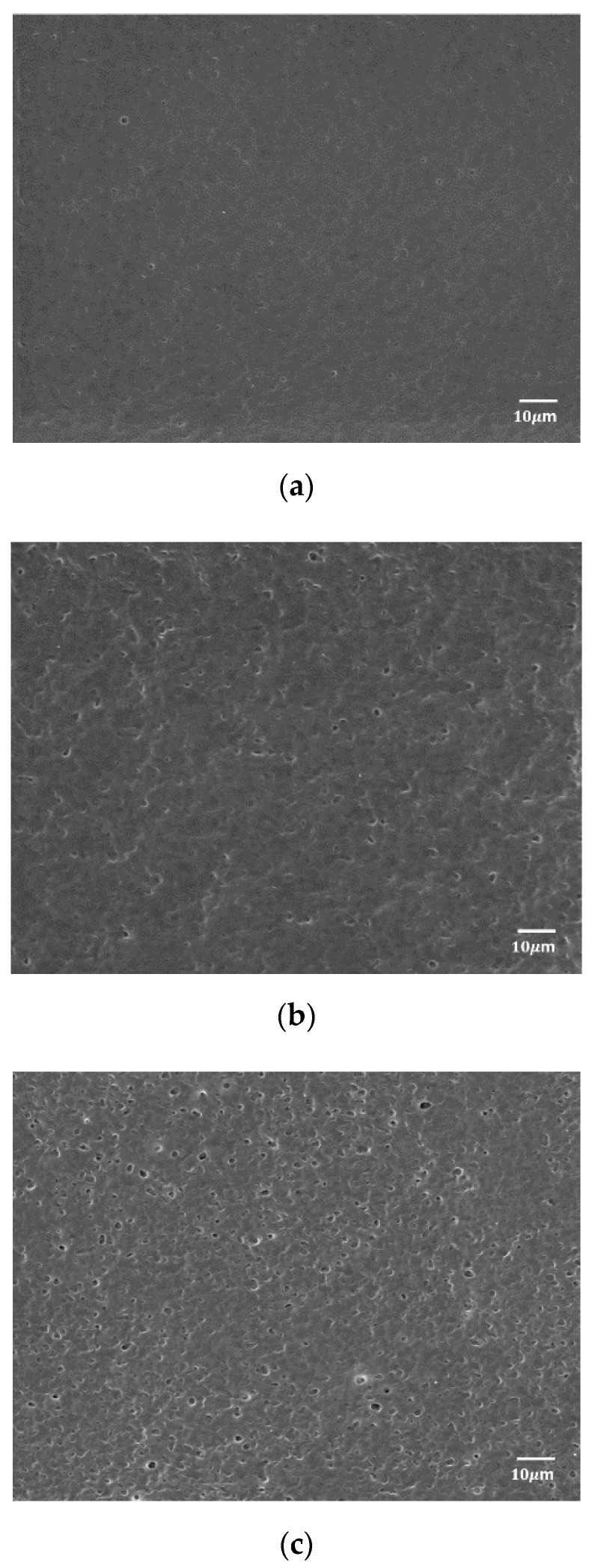
SEM images of (**a**) neat CA, (**b**) 1:0.23 CA/Ni(NO_3_)_2_·6H_2_O film dissolved in acetone/water (*wt/wt* 8:2) before applying a water pressure treatment and (**c**) 1:0.23 CA/Ni(NO_3_)_2_·6H_2_O film dissolved in acetone/water (*wt/wt* 8:2) after water treatment at 8 bar.

Scanning electron microscopy (SEM, JEOL JSM-5600LV, JEOL, Tokyo, Japan) was performed to observe the pores on the upper side of the CA membrane. Figure 2a–c shows the neat CA (Mn ~50,000) without water pressure treatment, 1:0.23 CA/Ni(NO_3_)_2_·6H_2_O without hydraulic pressure exposure and 1:0.23 CA/Ni(NO_3_)_2_·6H_2_O membrane after application of a water pressure treatment at 8 bar, respectively. In Figure 2c, the presence of fine pores sized 3–4 μm could be confirmed by observing the surface of the membrane that was subjected to the hydraulic pressure; pore formation led to an increase in the surface area of the membrane. From these results, it was anticipated that the hydrophilic impurities could be well adsorbed through the membrane pores.

**Figure 3:**
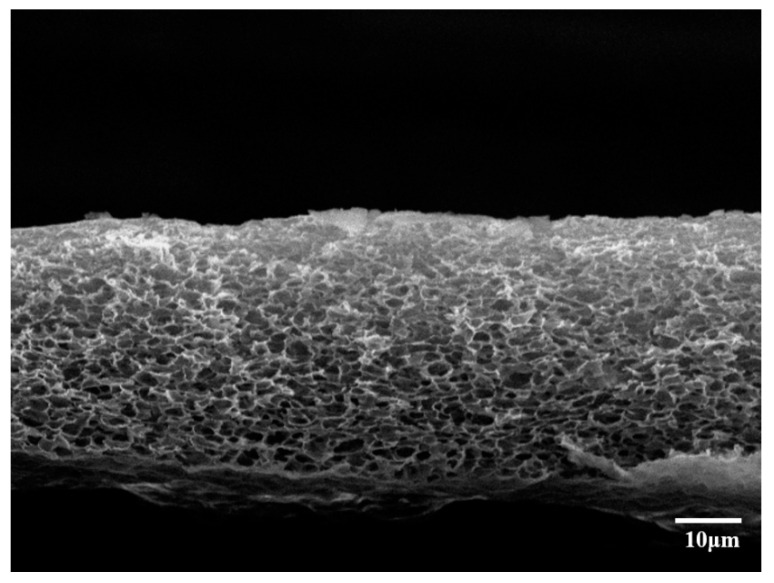
SEM images of 1:0.23 CA/Ni(NO_3_)_2_·6H_2_O film dissolved in acetone/water (*wt/wt* 8:2) after water treatment at 8 bar.

Figure 3 shows a cross section of the 1:0.23 CA/Ni(NO_3_)_2_·6H_2_O membrane after water treatment at 8 bar. The cross section shows a porous morphology that looks sponge-like. The size of the pores distributed in the membrane is about 2–4 μm, and the thickness of the membrane was measured at approximately 35 μm.

### 3.3. Fourier-Transform Infrared (FT-IR) Spectroscopy

**Figure 4:**
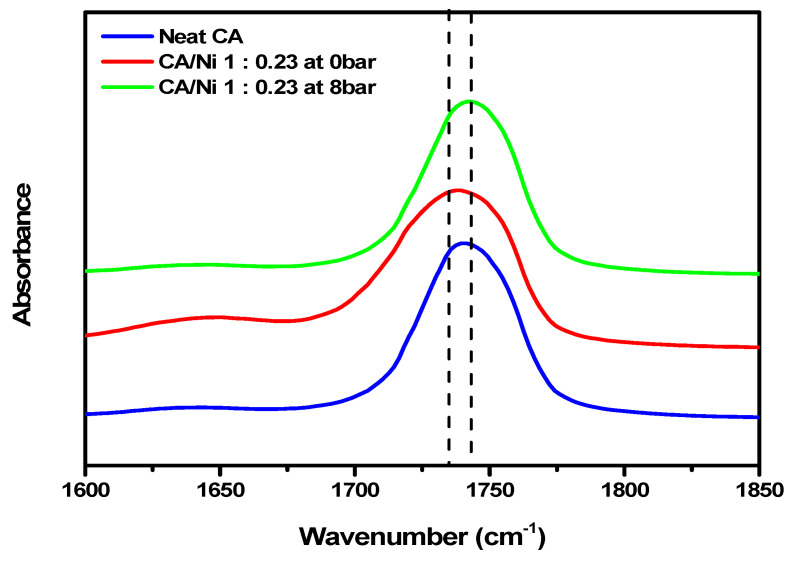
FT-IR spectra of neat CA, 1:0.23 CA/Ni salt at 0 bar and 1:0.23 CA/Ni salt at 8 bar.

FT-IR spectroscopy was used to investigate the interaction between the carbonyl groups of CA and the nickel salt. Figure 4, which compares the carbonyl peaks of neat CA and 1:0.23 CA/Ni salt at 0 bar, shows that the peak shifted from 1741 to 1737 cm^−1^ and broadened. The carbonyl peak of CA before applying water pressure was 1737 cm^−1^, which shifted to 1741 cm^−1^ after applying water pressure to the membrane. Therefore, it was demonstrated that most of the nickel(II) nitrate hexahydrate salt was removed after the pores were formed upon application of the water pressure.

**Figure 5:**
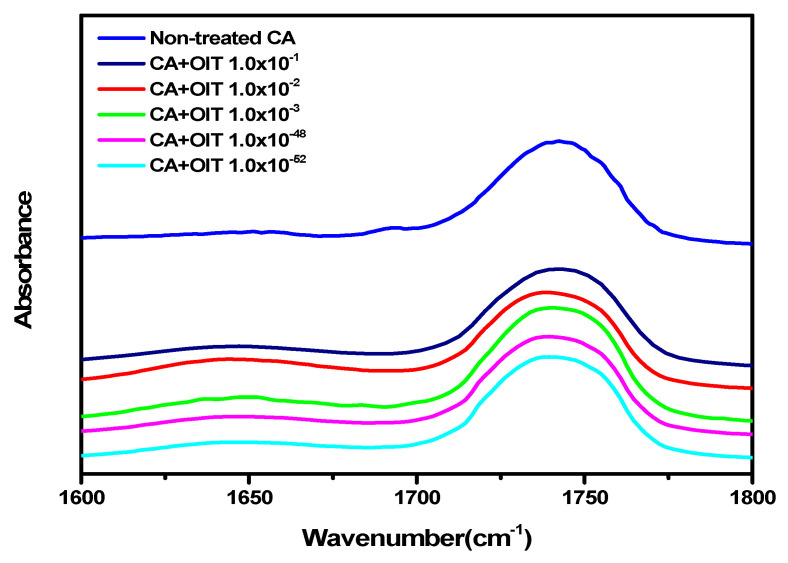
FT-IR spectra for the interaction between the carbonyl groups of the cellulose acetate (CA) polymers with 2-n-octyl-4-isothiazolin-3-one (OIT) at various concentrations.

**Figure 6:**
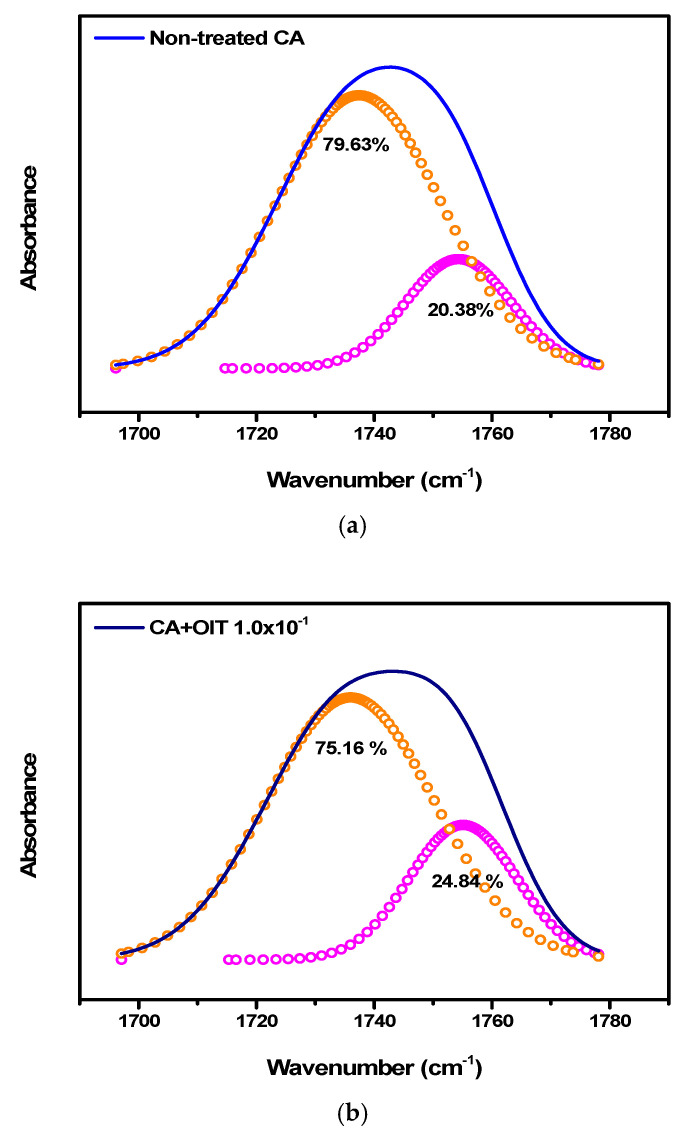

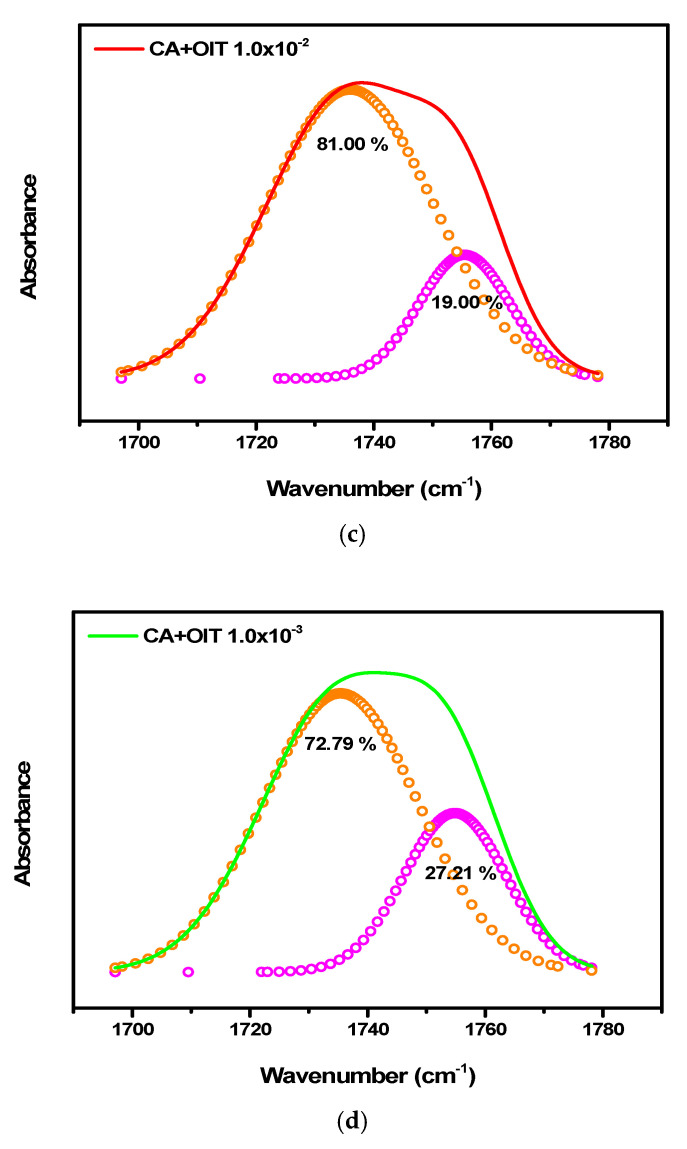
Deconvolution spectra of (**a**) neat CA, (**b**) 1:1.0 × 10^−1^ CA/OIT, (**c**) 1:10 × 10^−2^ CA/OIT and (**d**) 1:10 × 10^−3^ CA/OIT.

As can be seen in Figure 5, when an OIT sample was adsorbed on the 1:0.23 CA/Ni salt membrane at 8 bar, the carbonyl peaks changed either slightly or not at all. When comparing the peaks within the region ranging from 1735 to 1737 cm^−1^ in Figure 6 and Table 2, a very small area variation of 1.37–6.84% was observed for OIT concentrations of 1.0 × 10^−1^–1.0 × 10^−3^. Therefore, since this membrane did not strongly interact with OIT, it can be expected that OIT would easily pass through the column without being adsorbed when using it as a pretreatment membrane for sensing.

### 3.4. Thermogravimetric Analysis (TGA)

**Figure 7:**
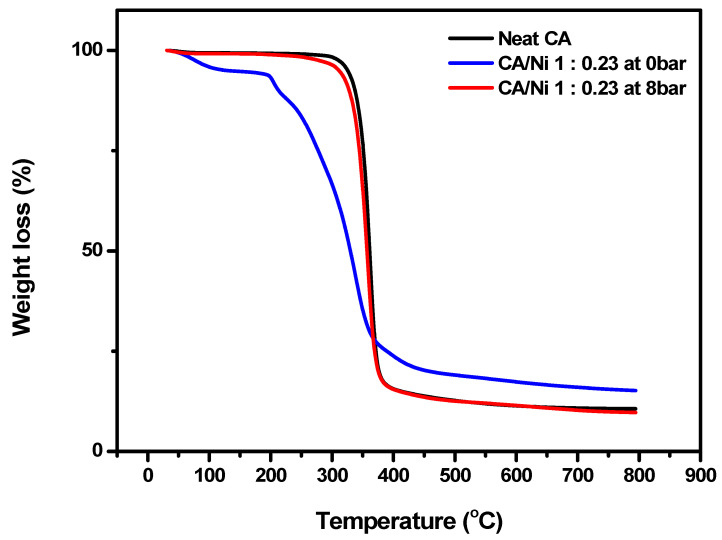
Thermogravimetric curves of neat CA, 1:0.23 CA/Ni(NO_3_)_2_·6H_2_O at 0 bar and 1:0.23 CA/Ni(NO_3_)_2_·6H_2_O at 8 bar.

In Figure 7, TGA was conducted to explore the plasticization of the polymer matrix and further compare the thermal weight loss before and after the addition of the nickel salt to the membrane, as well as before and after applying the water pressure. When comparing the weight loss of neat CA and 1:0.23 CA/Ni salt at 0 bar, the degradation temperature of neat CA was approximately 340 °C, while 1:0.23 CA/Ni salt at 0 bar exhibited a weight loss at approximately 240 °C due to a plasticization effect caused by the nickel(II) nitrate hexahydrate salt. After applying the water pressure, 1:0.23 CA/Ni salt at 8 bar underwent a weight loss at approximately 340 °C, similar to neat CA. Subsequently, water pressure treatment resulted in the formation of pores, and it was confirmed that most of the nickel salt was released.

## 4. Conclusions

In this study, the suitability of a CA-based porous membrane as a pretreatment filter device for the detection of OIT was investigated. The CA polymer is an environment-friendly, low-cost and energy-efficient material. Moreover, CA possesses many hydrophilic functional groups, such as carbonyl and ether groups. By exploiting this feature, hydrophilic impurities can be adsorbed onto the membrane, while hydrophobic substances like OIT can be expected to easily pass through a CA membrane column.

Since the interaction between OIT and the pretreatment membrane was found to be very weak, when OIT mixed with hydrophilic impurities was passed through the membrane, the hydrophilic substances were captured within the CA pores, while OIT easily reached the detection device. FT-IR spectroscopic analysis was used to confirm the extent of OIT adsorption on a 1:0.23 CA/Ni salt membrane after application of the water pressure. In Table 2, since the area difference of the deconvoluted data was closely observed, it could be suggested that the OIT and membrane formed a weak bond. Furthermore, a water uptake experiment was conducted to determine the extent of binding with the hydrophilic groups. It can be concluded that the hydrophilic materials were adsorbed after approximately 6 h, indicating that the membrane could strongly bind with these substances. As a result, the CA membrane column developed herein is suitable as a pretreatment film, since it could adsorb the hydrophilic impurities to a satisfactory extent, while allowing the hydrophobic OIT through.

## Figures and Tables

**Table 1:** Water uptake data.

Time (h)	Water Uptake (%)
0	100
2	45.95
4	9.75
6	1.60

**Table 2:** Area ratio of deconvoluted carbonyl peaks at various OIT concentrations.

Peaks	Neat CA	CA/OIT 1:1.0 × 10^−1^	CA/OIT 1:1.0 × 10^−2^	CA/OIT 1:1.0 × 10^−3^
1735–1737 cm^−1^	79.63%	75.16%	81.00%	72.79%
1754–1755 cm^−1^	20.37%	24.84%	19.00%	27.21%
